# Valorization of Moroccan Bentonite Deposits: “Purification and Treatment of Margin by the Adsorption Process”

**DOI:** 10.3390/molecules26185528

**Published:** 2021-09-12

**Authors:** Hanane Ait Hmeid, Mustapha Akodad, Mourad Baghour, Abdelmajid Moumen, Ali Skalli, Ghizlane Azizi, Hicham Gueddari, Mostapha Maach, Mimoun Aalaoul, Ahmed Anjjar, Lahcen Daoudi

**Affiliations:** 1 Laboratory Observatory of the Marchica Lagoon of Nador and Limiting Regions (OLMAN-RL), Multidisciplinary Faculty of Nador, Mohamed 1st University, Nador 60700, Morocco; akodadmfpn@hotmail.fr (M.A.); mbaghour@hotmail.com (M.B.); abelm127@hotmail.com (A.M.); all_skalli@yahoo.es (A.S.); ghizlaneazizi@hotmail.com (G.A.); hichamgueddari92@gmail.com (H.G.); Mostaphamaach12@gmail.com (M.M.); 2 Laboratory of Applied Geosciences, Faculty of Sciences of Oujda, Mohamed 1st University, Oujda 60000, Morocco; aa_mimoun@hotmail.com; 3 Laboratory of Georessources and Environment (GRE), Faculty of Science and Technology, University Sidi Mohamed Ben Abdellah, B.P.2202-Imouzzer Road, Fes 30000, Morocco; anjjar01ahmed@gmail.com; 4 Laboratory of Geosciences Georesources and Environment, Department of Geology, Faculty of Science and Technology University Cadi Ayyad, Marrakech 40000, Morocco; l.daoudi@uca.ac.ma

**Keywords:** raw bentonites, retention, margin, heavy metal, adsorption, phenolic compounds

## Abstract

The main objective of this work was to contribute to the reduction in the contamination of phenolic compounds contained in margin by an adsorption process on two types of raw bentonite. The margin used in the studies was collected from a semi-modern oil mill located in the Nador–Morocco region. The results of the physico-chemical analyses showed that the effluents of the oil mills showed that they are highly polluted, particularly in terms of the total suspended solids (TSS), chemical oxygen demand (COD), and iron content of around 154.82 (mg/L), and copper content of 31.72 (mg/L). The mineralogy of bentonites studied by X-ray diffraction (XRD) reveals the existence of two types of montmorillonite; theoretically, the diffraction peak (001) of the montmorillonite appears at 15 Å, with a basal spacing that corresponds to a calcium pole, and the diffraction peak (001) appears at 12Å, with a basal spacing that corresponds to a sodium pole. The specific surface area of the bentonite used is characterized by a large specific surface area, varying between 127.62 m^2^·g^−1^ and 693.04 m^2^·g^−1^, which is due to the presence of hydrated interleaved cations. This surface is likely to increase in aqueous solution depending on the solid/liquid ratio that modulates the degree of hydration. With a high cation exchange capacity (CEC) (146.54 meq/100 g), samples of margin mixed with raw bentonites at different percentages vary between 5% and 100%. The potential of Moroccan bentonite for the phenol adsorption of 9.17 (g/L) from aqueous solutions was investigated. Adsorption tests have confirmed the effectiveness of these natural minerals in reducing phenolic compounds ranging from 8.72% to 76.23% contained in the margin and the efficiency of heavy metal retention through microelements on raw bentonites. The very encouraging results obtained in this work could aid in the application of adsorption for the treatment of margin.

## 1. Introduction

Oil mills are among the industries with significant pollution emissions following the extraction of olive oil [1]. Morocco, characterized by a wealth of natural resources, is among the Mediterranean countries with the highest production of olive oil [2,3]. This industry, so beneficial for the national economy, leaves polluting and toxic liquid discharges (margin) [4]. Margin is also characterized by a high concentration of organic matter, which is poorly biodegradable and highly toxic [5]. They consist of water (83% to 92%), organic compounds (4% to 16%), and inorganic compounds (1% to 2%) [6]. These liquid discharges from oil mills must not be discharged directly into the sewage system. In this context, various studies have been carried out to eliminate phenolic compounds before they are discharged to the receiving environment [2,3,7,8,9]. Adsorption is one of the techniques that are widely used for the removal of pollutants which can occur between a solid and a gas or between a solid and a liquid [10,11]. It can be defined as the phenomenon of fixation of atoms or molecules on the surface of the solid by weak interaction forces of van der Waals type [12]. It is thus the passage from the dissolved to the adsorbed state. The opposite process is desorption [13]. Adsorption is caused by charged sites on the surface of the adsorbent, and the adsorption capacity is directly related to the number of these sites per unit area [14]. Bentonites can be used as adsorbents to respond to environmental problems [14,15,16].

This use has become one of the most important applications of bentonite in Europe and America, although in Africa, due to its abundance in nature and its low cost compared to other adsorbent materials, bentonite is considered to be a crucial element, especially in the adsorption process [2]. This study proposes the physico-chemical characterization of the liquid wastes from oil mills and to study the feasibility and efficiency of the treatment of phenolic compounds by adsorption with respect to two Moroccan crude bentonites, Trebia and Iboughrdayn. Additionally, we reflect upon the characterization of raw bentonites (without modification).

## 2. Results and Discussion

### 2.1. Characterization of Adsorbent

#### 2.1.1. Physicochemical Properties

Table 1 provides the results of the physicochemical properties of the bentonites used, such as the adsorbent. Bentonite of Trebia is characterized by the clay fraction (<2 μm) in the order of 9.09%, the silt fraction from 58.69% and the sand fraction from 32.20% [17]. Moreover, Bentonite of Iboughrdayn presents a clay fraction from 17.3, the silt fraction from 38.7 and the silt fraction 43.9%. All samples indicate mild alkalinity with pH values between 8.7 and 9.12 due to the presence of alkali carbonates and bicarbonates or silicates. These soluble and basic salts are generally associated with the composition of clay. The surface area of the bentonites is very important; we deduce that it is characterized by a significant amount of Smectite [17,18]. The free swelling index of bentonites varies between 36.53% and 59.18%, suggesting that the bentonites used have significant swelling properties.

#### 2.1.2. XRD Characterization

**Figure 1:**
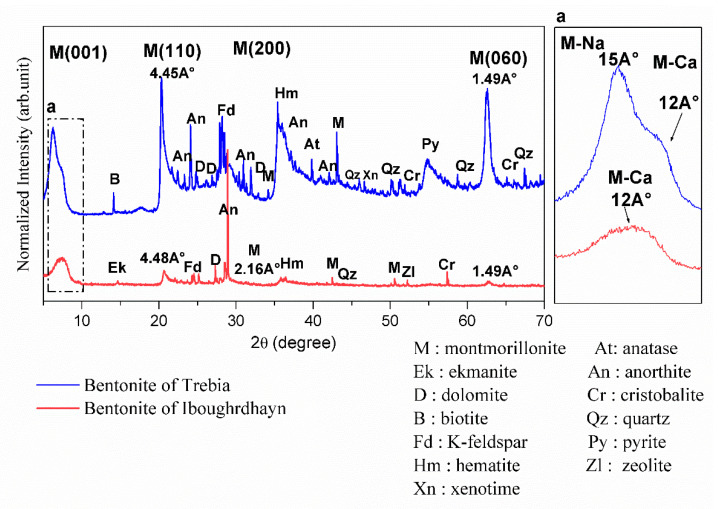
X-ray diffractograms of the raw bentonite samples.

The results of the XRD on the disoriented powder of the bentonite samples are shown in Figure 1. XRD analysis of raw Iboughrdayn bentonite shows the presence of montmorillonite, with the first peak located at a spacing of 12Å at d_001_ reflection; this shows that the natural bentonite is in the form of calcium, and the other three peaks are located at 4.45Å (d_110_), 2.567 Å (d_200_), and 1.49 Å (d_060_). The d_060_ reflection indicates the dioctahedral character of the smectite [19,20]. The plagioclase feldspar group with a calcium pole is manifested by anorthite peaks and orthoclase (K-feldspar). It is characterized by a low amount of hematite, quartz, ekmanite, dolomite, zeolite, and cristobalite. In the Trebia deposit, bulk mineralogy is characterized by the association of two types of montmorillonite; the first diffraction peaks (d_001_) of montmorillonite occur at a basal spacing of 15Å and 12Å. Theoretically, the peak at 12Å (d_001_) corresponds to a calcium pole (M-Ca) and the peak at 15Å (d_001_) corresponds to a sodium pole (M-Na). The other three peaks are located at 4.48 Å (d_110_), 2.16Å (d_200_), and 1.49 Å (d_060_). A moderate amount of K-feldspar, hematite, anorthite, and cristobalite; and secondary minerals quartz, anatase, dolomite was found, with traces of pyrite, zeolite, and xenotime.

#### 2.1.3. Spectrum Analysis

**Figure 2:**
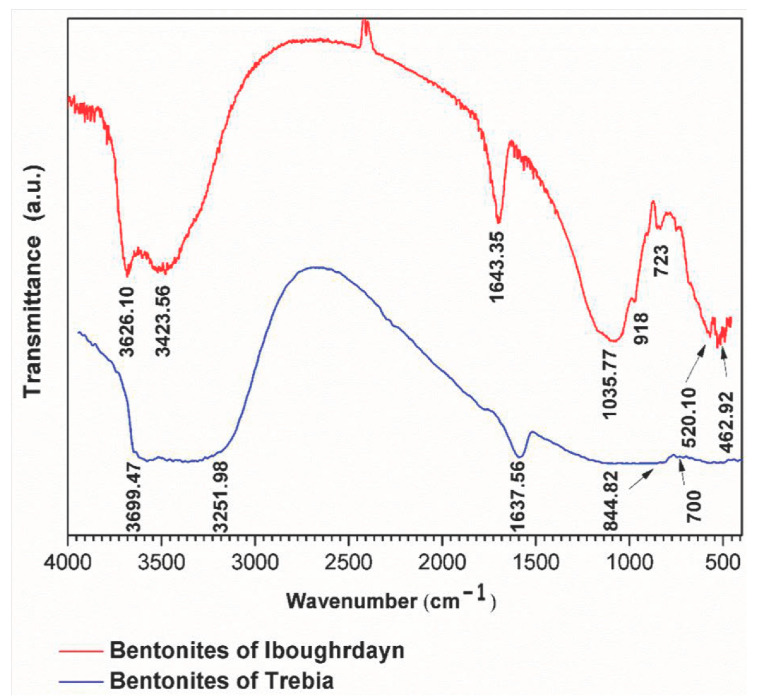
FTIR spectra of raw bentonite studied.

The FTIR analyses of the raw bentonite samples were taken in the range of 400–4000 cm^−^^1^ (Figure 2). The sample of Iboughrdayn is characterized by very strong multiple absorption of montmorillonite [21], observed in the range of 3626.10 to 3423.65 cm^−^^1^; these bands are attributed to the elongation vibrations of the binding of internal O-H groups in the structure of montmorillonite [ν OH] at the octahedral layer. As well as the peaks produced at around 3626 cm^−^^1^, this probably shows the presence of magnesium in the structure of the analyzed bentonites [22,23,24,25]. The spectrum of Terbia’s bentonite exhibits an absorption band at 3699.47 cm^−^^1^, assigned to the stretching and bending vibrations of the OH groups for the water molecule of hydration, which adsorbed on the bentonite surface [26]. This was followed by a large band at 3251.98 cm^−^^1^ in the mineral, attributed to OH stretching (ν_3_) the structural hydroxyl groups and water present in the mineral. This indicates the possibility of a hydroxyl bond between the octahedral and tetrahedral layers [27]. The intense band present in the range between 1635 and 1637 cm^−^^1^ corresponds to the asymmetric (ν_2_) OH (deformation mode) of the interlayer water and a structural part of the mineral [27,28,29]. Moreover, Iboughrdayn bentonite’s very strong absorption band observed at 1035.77 cm^−^^1^ is recognized for its stretching vibration Si-O bands, which are characteristic of stratified montmorillonite silicate mineral and attributed to the triple degenerate extension Si-O ν_3_ (in the plane) [30]. The presence of a band at 844.82 cm^−^^1^ in all samples is attributed to Al-Mg-OH deformations [31,32]. The bands located at 700 cm^−^^1^ and 723 cm^−^^1^ indicate Si-O stretching due to the presence of quartz [33,34]. The bands are at 520 for Si-O-Al (octahedral) and 462 cm^−^^1^ for Si-O-Si bending vibrations.

#### 2.1.4. XRF Characterization

The chemical constitution of the adsorbents is represented in Table 2. The bentonite samples are characterized by a large amount of Fe, between 28.6% and 30.93%, related to iron ore such as hematite (Fe_2_O_3_), identified by both XRD analysis and FTIR spectra. The silice content, from 0.1% to 7.94%, is related to quartz, cristobalite and feldspar. Samples are characterized by a medium content of calcium, ranging from 11.1% to 14.01%, and the aluminum content, between 4.81% to 7.3%, which are part of the mineralogical composition of montmorillonite. Magnesium was identified in the samples, although Al^3+^ ions can be partially substituted by Mg^2+^ ions in the octahedral layers, which explains the decrease in aluminum content in the bentonite of Iboughrdayn offset by magnesium [35].

### 2.2. Characteristics of Raw Margin

The margin sample was analyzed prior to treatment for different physico-chemical properties, and the results of the analysis are presented in Table 3. These values represent the averages of three determinations. According to the compositions of these raw margins, it appears that it is a highly polluting and harmful organic effluent of an organic nature. It is worth noting that the parameter values are in good agreement with those reported in the literature. It can be seen that the pH of margin is relatively low (pH = 4.03). This is mainly due to the presence of compounds such as organic acids and phenols. Indeed, the recorded electrical conductivity is relatively average, in the order of 16.93 ms·cm^−^^1^. The margin used contained fewer suspended solids of 5.93 g·L^−^^1^. Organic matter expressed in COD has a very high value of the order 172, 72 g·L^−^^1^. A high content of compounds such as phenolic acids and fatty acids was found, with total polyphenols present at a rate of almost 9.17 g·L^−^^1^. The concentrations of nitrogen elements (TKN: 2.39 g·L^−^^1^), and phosphate elements (total phosphorus: 1.16 mg·L^−^^1^) are relatively high.

### 2.3. Adsorption Experiments

#### 2.3.1. Evolution of Physico-Chemical Parameters

**Figure 3:**
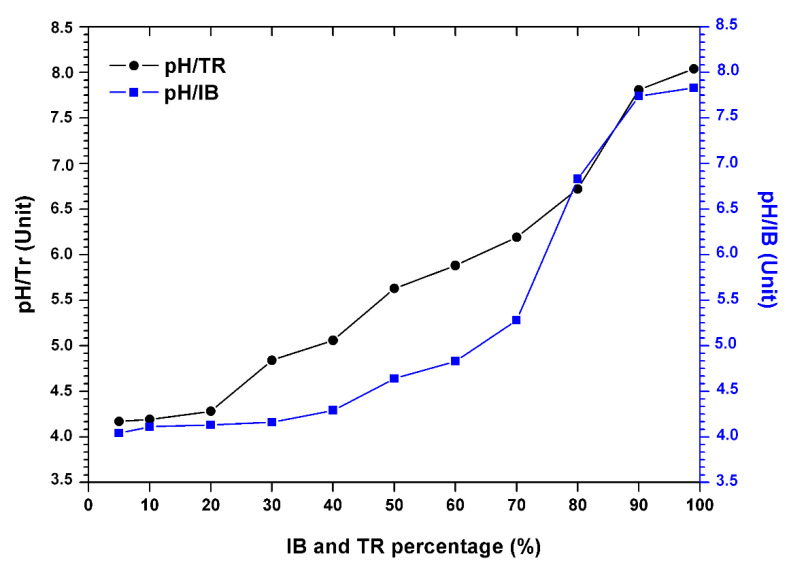
Evolution of the pH of the margin–bentonite mixtures.

The results in Figure 3 show the variation in the pH of the bentonite/margin mixture as a function of the increase in the percentage of bentonites.

There is an increase in pH (Table 4). However, the acid pH varies between 4.04 and 4.17, at the alkaline pH from 7.83 to 8.04 between 5% and 80% of the concentration of bentonite.

Still, a sudden increase in pH was noted when the percentage of bentonite was over 80%. This increase is probably related to the composition of the bentonite which is rich in soluble and basic salts such as alkali carbonates and bicarbonates or silicates, which have a strong capacity to neutralize the acidic pH of the mixture.

At a percentage of 90%, Trebia bentonite tends to neutralize the pH value of the 7.81 mixture. In addition, for Iboughrdayn bentonite, the average pH value is around 7.74.

**Figure 4:**
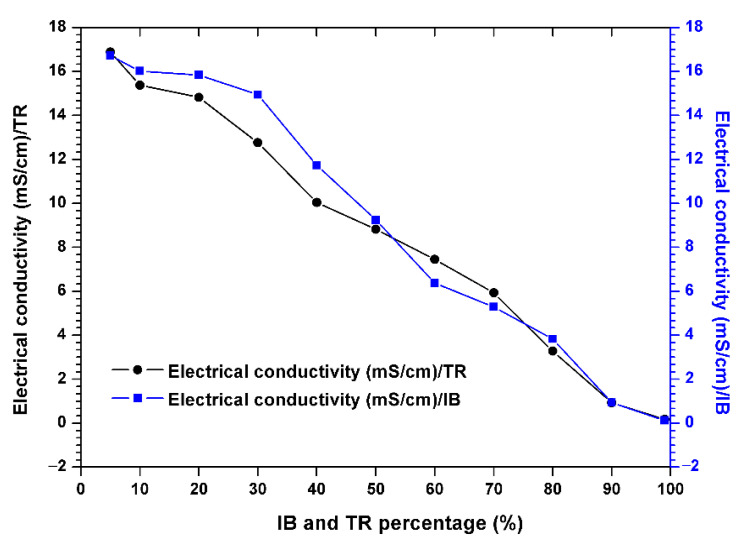
Evolution of the electrical conductivity of the margin–bentonite mixtures.

Figure 4 shows the electrical conductivity, showing that this parameter decreases steadily throughout the experimental period. The passage of the raw margin through the mixed margin shows a significant and successive decrease in electrical conductivity in the Trebia and Iboughrdayn mixtures, from 16.88–16.72 to 0.17–0.12 µS/cm.

#### 2.3.2. Total Suspended Solid Removal

**Figure 5:**
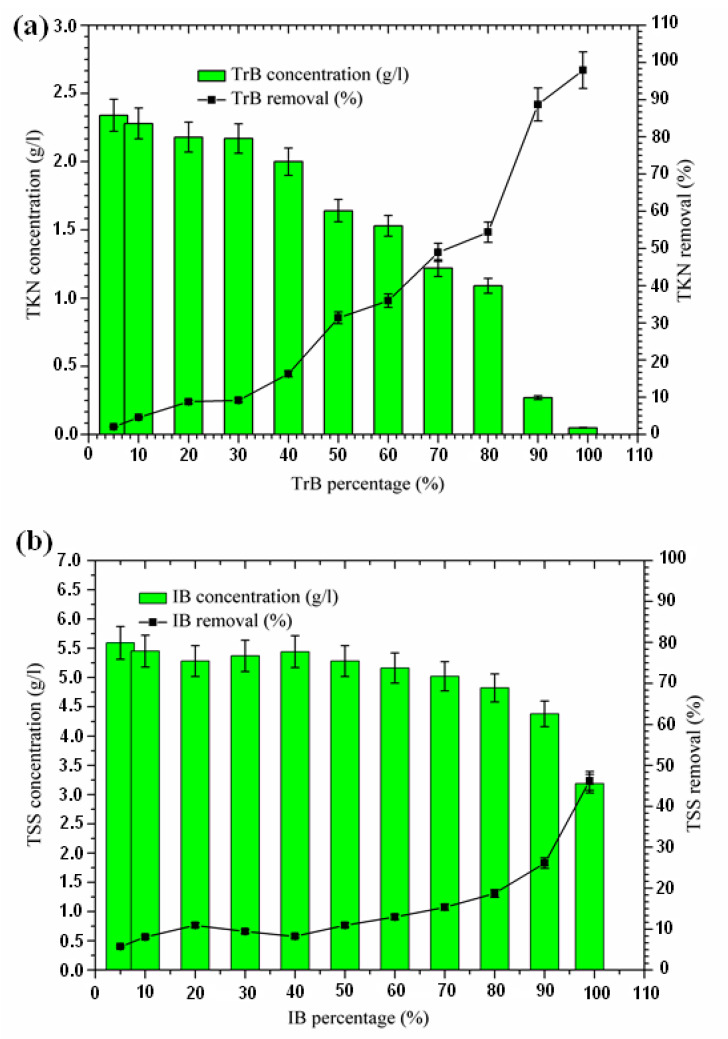
(**a**) Concentration and removal percentage of TSS by TrB; (**b**) concentration and removal percentage of TSS by IB.

The influence of adsorbent dose on the adsorption of total suspended solids from the margin is shown in Figure 5. An increase in suspended solid removal with an increase in adsorbent dose was recorded, starting at a concentration of 50%. The TSS concentration for the Iboughrdayn bentonite mixture ranges from 3.19 to 5.59 g·L^−^^1^. In combination, the TSS concentration for Trebia bentonite decreased from 5.88 g·L^−^^1^ to 3.82 g·L^−^^1^. Trebia’s bentonite corresponds to a reduction of 35.58%. Iboughrdayn bentonite has a reduction rate of 46.21%. The variation in the rate of reduction of TSS can be explained by the particle size composition of the bentonites (Table 1). Therefore, the bentonite mixture plays the role of the primary barrier, which led to a reduction in TSS.

#### 2.3.3. Organic Matter Reduction

**Figure 6:**
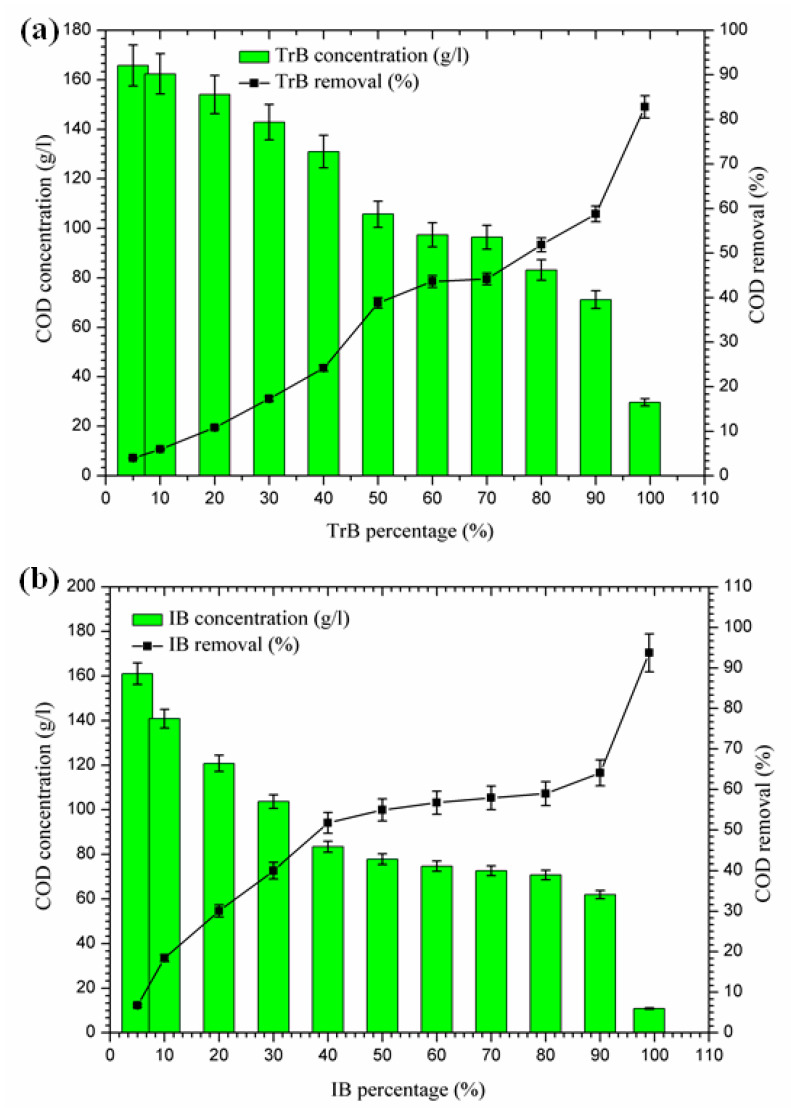
(**a**) Concentration and removal percentage of COD by TrB; (**b**) concentration and removal percentage of COD by IB.

Figure 6 shows a significant reduction in COD, in contrast to Bentonite of Terbia and Iboughrdayn, which varies between 82.80 and 93.73%. This reduction is due to the degradation of organic matter by bentonite. Figure 6 shows the variation in total COD concentration and the rate of reduction as a function of the percentage of bentonite. The evolution of this parameter was divided into three distinct phases:

- The first phase (5–50%): where the COD concentrations for the margin mixture with Iboughrdayn bentonite were reduced from 161.03 to 77.83 g·L^−^^1^. At the same time, we noticed an increase in the COD removal rate from 6.77% to 54.94%. In addition, Trebia margin/bentonite, characterized by reduced concentrations from 165.81 to 105.70 g·L^−^^1^, corresponds to elimination rates from 4.00% to 38.80%;
- The second phase (50–70%): the COD concentrations for the almost constant margin/bentonite mixture vary successively for the Iboughrdain and Trebia bentonite in the interval between 73 and 96.5 g·L^−^^1^. In addition, COD removal rates vary between 57% and 44%;
- The third phase beyond 70%: we distinguished a remarkable increase at the end of the treatment of the rate of elimination, with Iboughrdayn and Trebia bentonite reaching up to 93.73% and 82.80%, respectively.

Then, the reduction in the COD rate is probably due to the reduced decomposition of organic matter affected by the formation of the adsorption conditions [34].

In fact, the decrease in COD is related to the reduction in organic matter decomposition, which is now adsorbed on the bentonite, forming the organoclay complex. The bentonite in this complex plays a protective role against the decomposition of organic matter.

#### 2.3.4. Nitrogen Removal

**Figure 7:**
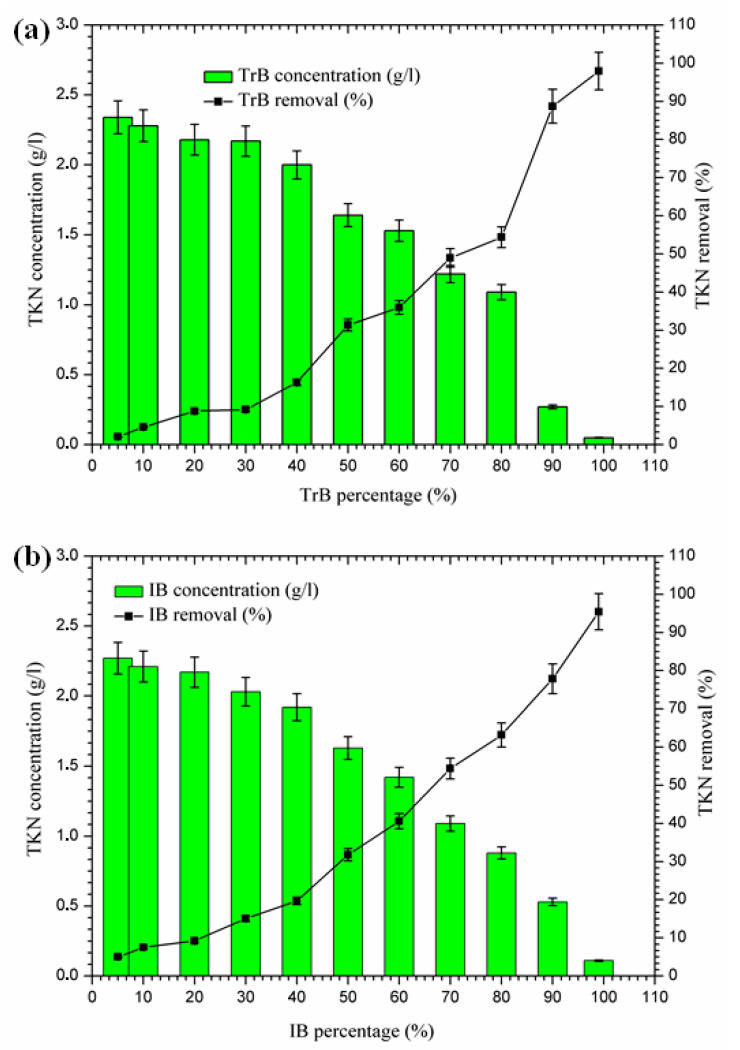
(**a**) Concentration and removal percentage of TKN by TrB; (**b**) concentration and removal percentage of TKN by IB.

The concentration of TKN nitrogen in Iboughrdayn’s bentonite-treated margin is of the order of 2.27 g·L^−^^1^, and decreases to 0.11 g·L^−^^1^, effectively reduced at a rate of 95.40%. However, Trebia bentonite can remove TKN nitrogen concentrations of from 2.34 to 0.05 g·L^−^^1^. At the beginning of the experiment, we recorded a percentage reduction of 2.09% in NTK nitrogen, which gradually increased to reach a maximum value of 97.91% (Figure 7). In fact, the increase in removal efficiency for the concentration of TKN nitrogen in the bentonite can be explained by the texture of the bentonites used for the development of aerobic conditions and the denitrification process, as well as the adsorption on the surface of the bentonite, due to their large specific surface area (Table 1).

#### 2.3.5. Phosphorus Removal

**Figure 8:**
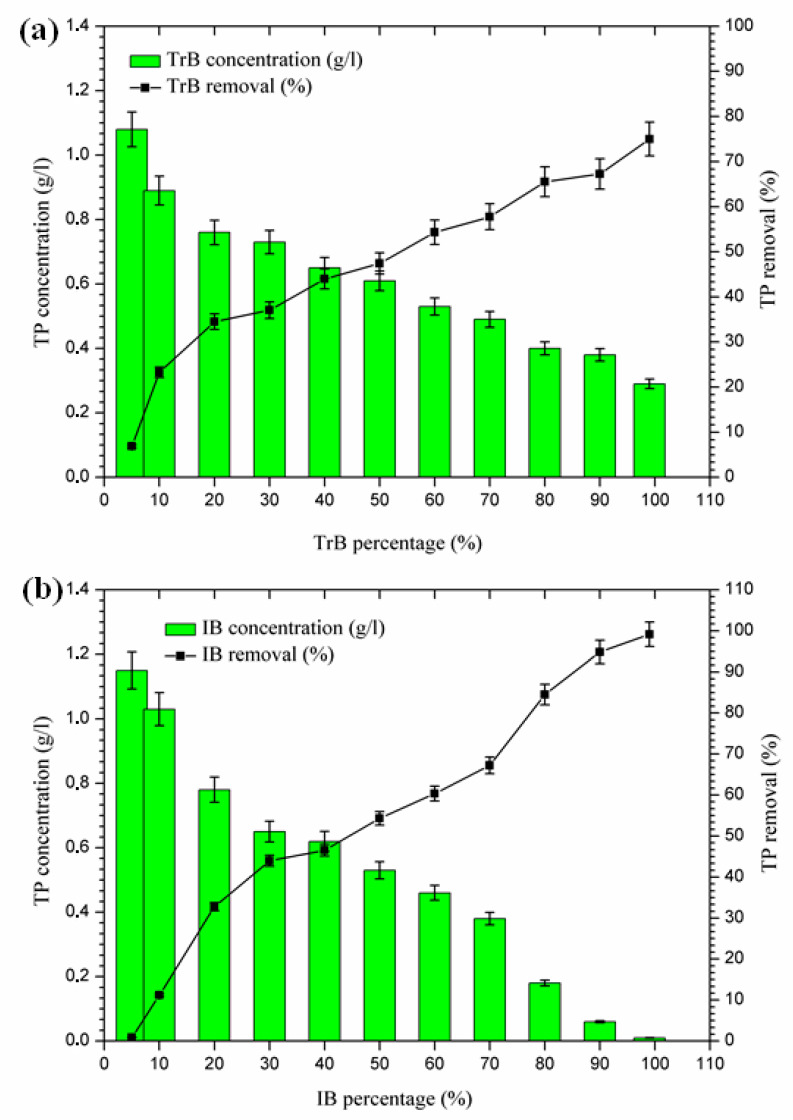
(**a**) Concentration and percentage of TP removed by TrB; (**b**) concentration and percentage of TP removed by IB.

According to the results presented in Figure 8, phosphorus removal occurs in two phases:

- The first Phase (5–20%): where the maximum phosphorus retention rate by Trebia bentonite, on the one hand, reached 34.48%, and on the other hand, Iboughrdayn bentonite at abatement reached 32.76%;
- The second phase (20–99%): where the maximum total phosphorus removal reaches values between 75% and 98.14%. Phosphorus can be removed by adsorption on iron and aluminum hydroxides (Al^3+^), by the precipitation process [36,37,38,39], which is indeed part of the chemical composition of bentonite (Table 2).

Therefore, the difference in the rate of phosphorus removal observed during treatment with Trebia bentonites and Iboughrdayn bentonite could be related to variations in the mineralogical composition and micromorphological and textural characteristics of these bentonites.

#### 2.3.6. Total Phenolic Compound Removal

**Figure 9:**
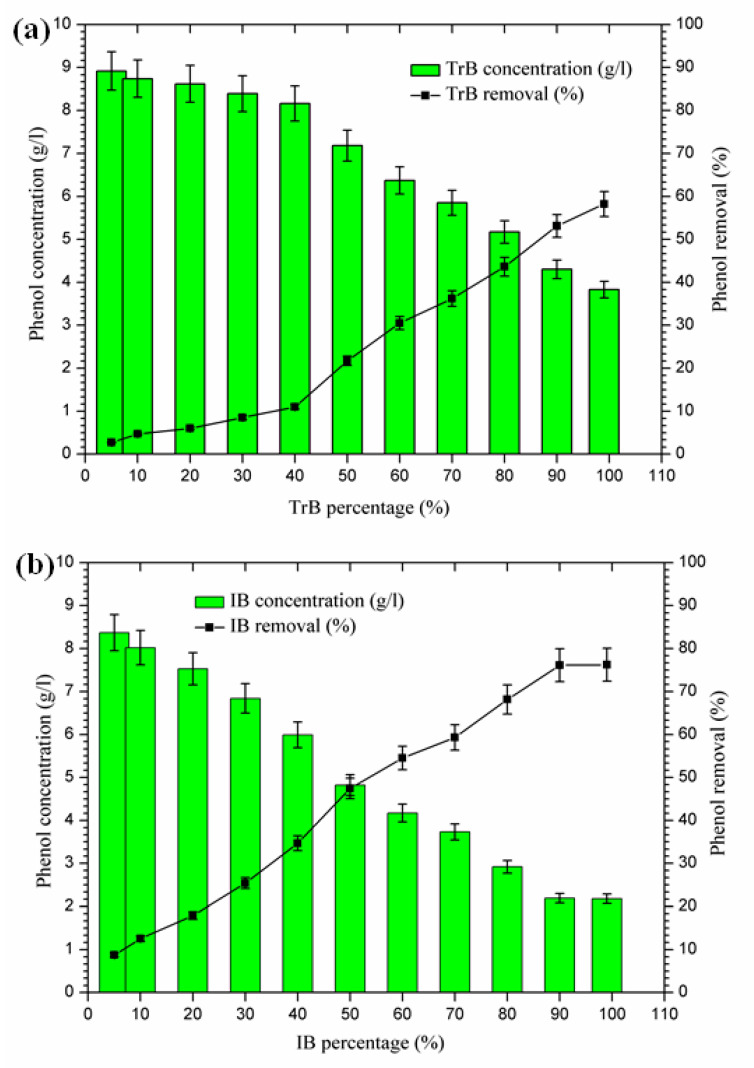
(**a**) Concentration and removal percentage of the phenol by TrB; (**b**) concentration and removal percentage of the phenol by IB.

The mixing of margin with Iboughrdayn bentonite at high rates of elimination of phenolic compounds (59.32–76.23%) was recorded for percentages between 70% and 99%, with a maximum observed when the contents of phenolic compounds were very low at the end of treatment. On the other hand, Trebia bentonite, with fairly average rates of elimination of phenolic compounds (30.53–58.23%), corresponds to percentages between 60% and 99%, with a maximum observed when the levels of phenolic compounds are very low, obtained at the end of treatment. However, the maximum removal rate was recorded at 60% bentonite (Figure 9).

The decrease in phenolic compounds would be related to the higher and/or increased pH [40,41]. Then, the phenolic compounds could be removed by adsorption on the surface of the bentonite, perhaps due to the higher dose of adsorbent providing more active adsorption sites or surfaces [9]. These data may explain the high removal rate of phenolic compounds found in the second phase (60% to 99%), where the percentages of bentonites and pH were significant.

#### 2.3.7. Variation of Microelements

**Figure 10:**
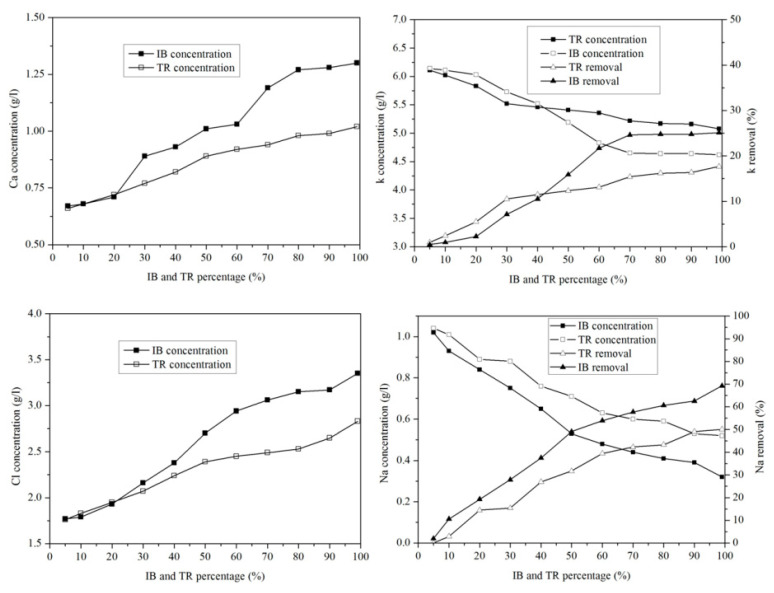
Concentration of and variation in micro-elements as a function of bentonite content.

Figure 10 shows the variation in microelements in the margin/bentonite mixture. The potassium concentration varies from 6.14 g·L^−^^1^ to 4.62 g·L^−^^1^ in the margin/bentonite mixture of Iboughrdayn. In comparison, the concentration of potassium decreases from 6.11 g·L^−^^1^ to 5.08 g·L^−^^1^ in the mixture of margin with Trebia bentonite, with removal rates varying in both the Trebia margin/bentonite mixture and the Iboughrdayn margin/bentonite mixture from 17.67% to 25.12%.

The mixture of margin with Trebia bentonite has sodium (Na) concentrations ranging from 1.04 g·L^−^^1^ to 0.60 g·L^−^^1^, corresponding to an elimination rate of the order of 42.31%. However, the mixture the margin with Iboughrdayn bentonite has sodium (Na) concentrations which decreased from 1.02 g·L^−1^ to 0.32 g·L^−1^. As well as this, we observed that the elimination rate of Na increased from 1.92% to 69.23%. Jointly, the concentrations of calcium (Ca) and chloride (Cl) increased with the increase in the percentage of bentonite. Indeed, the increase in these two elements is denoted from 20% onwards.

#### 2.3.8. Heavy Metal Ion Removal

In order to compare the adsorption potentials, raw bentonite from Trebia and Iboughrdayn was chosen and used for the optimal adsorption of metal ions from margin.

**Figure 11:**
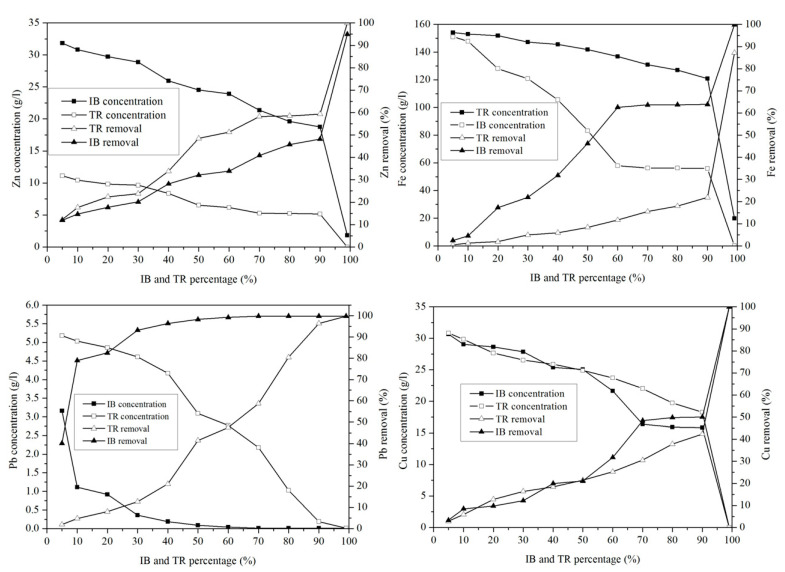
Concentration of and variation in heavy metals.

The concentration of Zn in the mixture of margin with Iboughrdayn bentonite varies between 31.72 g·L^−^^1^ and 0.01 g·L^−^^1^. The evolution of this element was split into two phases: in the first phase, percentages of the 90% lower bentonite correspond to rates between 11.93 and 48.12% (Figure 11). In the second phase, a sudden increase in the rate of elimination of Zn was observed at a maximum value of around 94.94%. In addition, the mixture of margin with Trebia bentonite was characterized by an ideal absorption power, where the concentrations recorded at the end of the experiment maintain that traces of Zn do not exceed 0.02 g·L^−^^1^.

This corresponds to a maximum rate of 99.97%. It was deduced that the adsorption of Pb occurs in a total way, where the elimination rate is denoted successively for Trebia and Iboughrdain bentonite from the percentages of 70% and 10%. Knowing this, the same Pb elimination rates were recorded in the order of 99.81%. The variation in the Cu adsorption in the two adsorbents obtains the same results, with an elimination rate of 99.97% Cu at percentage 99%, corresponding to concentrations of 0.01 g·L^−^^1^.

The decrease in the concentration of Fe in the mixture of margin with Iboughrdayn bentonite from 151.03 to 0.17 g·L^−^^1^ corresponds to a maximum rate of 99.89%. Moreover, the mixture of the curbstone with Trebia bentonite is characterized by an Fe removal rate of 87.19%.

### 2.4. Phenol Adsorption Mechanism on Bentonite

The adsorption process results from interactions between the surface of the adsorbent (bentonite) and the adsorbate (phenol, heavy metal, etc.).

The mechanisms responsible for the fixation of organic compounds on the bentonite material (formation of the organoclay complex) are multiple and are controlled by the properties of the adsorbent, particularly its specific surface, the value and location (tetrahedral or octahedral) of the electric charge, and the different characteristics of the organic adsorbate, of which the nature, size, structure, and basicity of the organic molecules are the dominant parameters.

**Figure 12:**
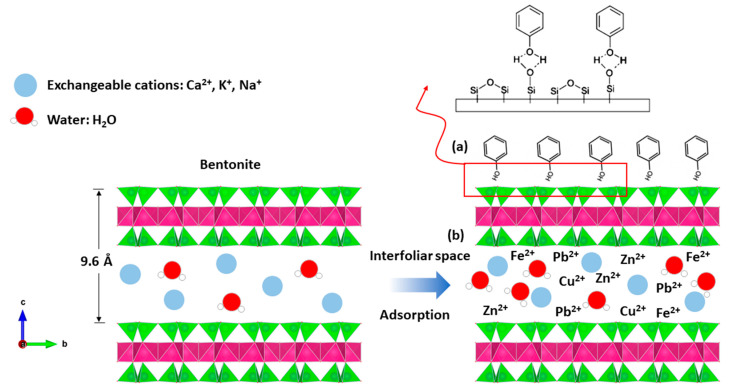
Adsorption mechanism between; (**a**) phenol and bentonite; (**b**) heavy metal and bentonite.

Indeed, bentonite treatment of margin increases the pH, which promotes the adsorption of phenols by attractive forces of chemical nature, causing a transfer or pooling of electrons and, consequently, the destruction of the individuality of molecules and formation of a chemical compound on the surface of bentonite (Figure 12a).

The bentonites studied have basic pH values (Table 1). The neutrality of the medium is probably related to various properties of bentonite material, including its high CEC; its adsorptive power; its composition, which is rich in basic salts such as carbonates, bicarbonates, oxides, hydroxides, etc.; and its ability to form an organo–mineral complex with certain organic compounds of the margin. This latter property plays a primordial role in the ionic fixation and increases the property of adsorption, bentonites’ excellent material of the depollution, etc., and so the heavy metals are easily introduced to the interfoliar space of the bentonite (Figure 12b).

## 3. Materials and Methods

### 3.1. Materials

**Figure 13:**
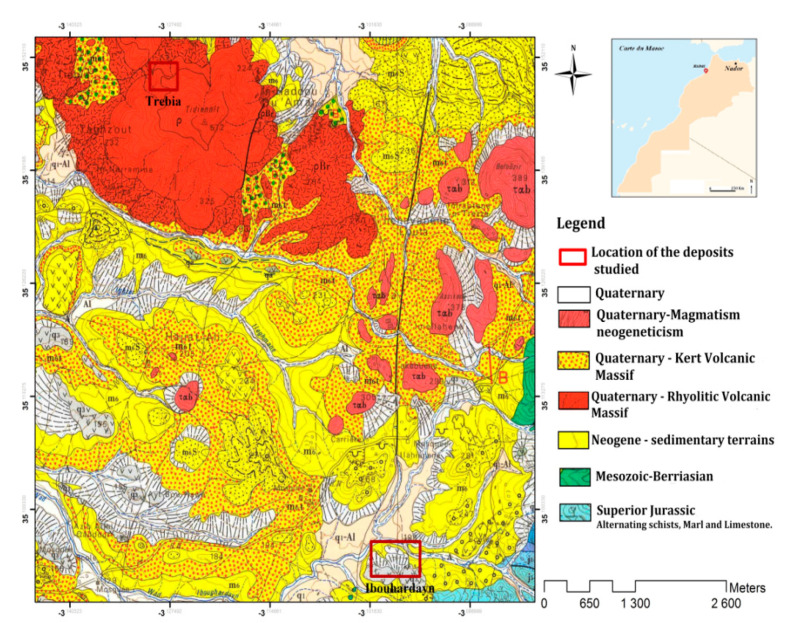
Geological map of the deposits studied.

The bentonite used in this work is distributed in the external domain of the Rifaine chain on the western flank of Nador city in the Kert neogenous basin (Figure 13). Bentonite comes from the Trebia and Iboughrdayn deposit in Nador (North-East Morocco). The elemental composition of the raw bentonite is illustrated in Table 1. The margin used in this study was collected from a semi-modern oil mill located in the province of Driouache, about 60 km from the city of Nador. The samples were taken during the olive oil extraction seasons (December–March) and during the olive-growing season 2018–2019. 

### 3.2. Preparation of the Adsorbent/Adsorbate

The experiments were performed in beakers with a capacity of 100 mL. For each test, the solution was mixed with the margin and a mass of the adsorbent (bentonites). The mixture was stirred permanently. To achieve the objectives of this study, we progressively substituted the bentonite contents with increased margin volumes. The percentages of applied margin were calculated in relation to the following mass loads: 5%, 10%, 20%, 30%, 40%, 50%, 60%, 70%, 80%, 90% and 99%.

### 3.3. Characterization Methods

The adsorbant samples were characterized by X-ray diffraction (XRD) (San Giovanni Valdarno (AR), Italy), Fourier transform infrared spectroscopy (FTIR) (Nador, Morocco), particle size distribution (Marrakech, Morocco), and chemical analyses (XRF) (Nador, Morocco). The crystal phases were determined by X-ray diffraction analysis, an XRD patterns were collected using an XPERT-PRO diffractometer (San Giovanni Valdarno (AR), Italy), operating at a voltage of 40 kV and an intensity of 30 mA with radiation (Cu), on the powdered sediments following the normal procedure for clay analysis [42]. Chemical analysis of the samples was determined using an X-ray fluorescence analyzer (Nador, Morocco). IR spectra were made using a Shimadzu FTIR-8400S spectrometer (Nador, Morocco) over a range of 400–4000 cm^−1^. The particle size distribution of the samples was analyzed on bulk sediment using a laser diffraction particle analyzer Horiba 300. Characterization of the physico-chemical parameters was performed by measuring acidity (pH), swelling index, and swelling rate.

The specific surface areas and the cation exchange capacity of the samples were found by the methylene blue test [43,44]. The values of the cation exchange capacity (CEC) were determined according to (1) [44]

CEC (meq/100g) = V_MB_ × N_MB_ × (100/m_0_) (1)

where V_MB_ = the volume of MB added to the sample; N_MB_ = the normality of methylene blue; m_0_ = the weight of the dry powdered sample.

### 3.4. Adsorption Methodology

The efficiency the margin treatment with bentonite was monitored by measuring the evolution of the physico-chemical parameters. The hydrogen potential (pH) of the mixture was determined by a pH meter according to the protocol described by Alkama et al. (2009) [45]. The electrical conductivity was measured using a Consort C933 conductivity meter. Suspended solids (SS) were determined by filtration on membranes with a pore diameter of 45 µm. The TSS content was determined by the difference in weight of the filter before and after filtration and drying in an oven at 105 °C for 4 h [46]. The determination of nitrogen (TKN) was carried out according to the Kjeldahl method described by Bargaz et al. (2012) [47]. The chemical oxygen demand (COD) was determined according to the standard method [48], with oxidation of the organic matter contained in the sample at 150 °C by an excess of potassium dichromate in an acidic medium and in the presence of silver sulphate. The excess potassium dichromate was determined by colorimetry at ʎ = 620 nm. Total phosphorus (TP) was determined according to standard methods by AFNOR (1997), APHA (2005), and Rodier (2009) [48]. This was measured after mineralization in acidic solution with the presence of sodium persulfate at 200 °C for two hours, followed by an analysis of the orthophosphates [49].

Total polyphenols were obtained according to the method recommended by Macheix et al. (1990) [50]. Total polyphenols were quantified from the 200 µL Folin–Cioccalteu reagent. They were then oxidized by blue oxides of tungsten and molybdenum, followed by 400 µL of a sodium carbonate solution (20%) [51,52]. The mixture was placed in an incubator at 40 °C for 30 min. Finally the absorbance reading was taken at 760 nm. The contents of phenolic compounds are expressed in g·L^−^^1^.

The contents of iron, copper, zinc, potassium, sodium, calcium, chloride, and lead were analyzed by atomic absorption spectrometry (model AFP100, Biotech Management Engineering Co. Ltd., Nicosia, Cyprus) [53,54].

Mass percent of the removal metal ion was calculated using the following Equation (2) [55].

% Removal = (C_0_ − C_f_/C_0_) × 100 (2)

where C_0_ and C_f_ (mg/L) are the initial and final equilibrium concentrations of solution.

## 4. Conclusions

The treatment and adsorption of margin on raw bentonite from Morocco was studied in an aqueous medium. This adsorption depends on the physicochemical parameters involved (pH, electrical conductivity, and mass of adsorbent). From all the results, obtained at the end of the application for adsorption by the raw bentonite, it could be concluded that the studied margins present a major risk for the environment. Therefore, the study of adsorption has allowed us to note that the efficiency of phenolic compounds’ removal from the copings by natural bentonite is important. This is explained by the presence of free sites on the surface of the particles of the adsorbent (bentonite), which correspond to the progressive decrease in concentrations. Given the very encouraging results which were obtained, we aim to study the application of adsorption for the treatment of margins. This method is simple, economical, ecological, non-toxic and fast, which is why we recommend its use for the treatment of domestic wastewater in developing countries.

## Figures and Tables

**Table 1:** Physicochemical properties of bentonite samples used in the study.

Sample	% Sand (>60 μm)	% Silts (2–60 μm)	% Clay (<2 μm)	IG (%)	C (%)	S_ST_ (m^2^/g)	CEC (meq/100 g)	pH
TrB	32.20	58.69	9.09	59.18	36	518.92	42.42	9.12
IB	43.9	38.7	17.3	36.53	45	630	127.12	8.7

Note: TrB: bentonite of Trebia; IB: bentonite of Iboughrdayn; S_ST_: specific surface area; CEC: cation exchange capacity; IG: swelling index; C: colloidality.

**Table 2:** Chemical composition of bentonite.

Weight (%)	IB	TrB
Ca	14.01	11.1
Fe	30.93	28.6
Al	4.81	7.3
Si	7.94	0.1
Mn	0.18	0.8
K	4.06	4.4
Sr	0.68	0.4
P	2.12	3.1
S	1.04	1.6
Th	0.033	0.1
Rb	ND	0.05
Y	0.033	0.02
Mg	32.1	29.3
Zn	0.55	0.08
Sn	ND	0.07
Ti	1.25	1.2
Pb	0.05	ND
Zr	0.18	0.3
As	ND	0.02

Note: ND; not detected.

**Table 3:** Physico-chemical characteristics of the raw margin.

Parameter	Unit	Mean ± Standard Deviation (*n* = 3)
pH	(Unit)	4.03
Electrical conductivity	(mS/cm)	16.93
Chemical oxygen demand	(g·L^−1^)	172.72
Total suspended solids	(g·L^−1^)	5.93
Total Kjeldahl nitrogen	(g·L^−1^)	2.39
Total polyphenols	(g·L^−1^)	9.17
Total phosphorus	(mg·L^−1^)	1.16
Micro-element concentrations (g·L^−1^)		
Ca	(g·L^−1^)	0.62
K	(g·L^−1^)	6.17
Na	(g·L^−1^)	1.04
Cl	(g·L^−1^)	1.72
Heavy metal concentrations (mg·L^−1^)		
Fe	(mg·L^−1^)	154.82
Cu	(mg·L^−1^)	31.72
Zn	(mg·L^−1^)	36.14
Pb	(mg·L^−1^)	5.28

**Table 4:** Evolution of the pH and the electrical conductivity of the margin-bentonite mixtures.

Sample	M_r_	Tr_5%_	Tr_10%_	Tr_20%_	Tr_30%_	Tr_40%_	Tr_50%_	Tr_60%_	Tr_70%_	Tr_80%_	Tr_90%_	Tr_99%_
pH (Unit)	4.03	4.17	4.19	4.28	4.84	5.06	5.63	5.88	6.19	6.72	7.81	8.04
EC (mS/cm)	16.93	16.88	15.37	14.82	12.76	10.04	8.82	7.45	5.92	3.28	0.93	0.17
**Sample**	**M_r_**	**Ib_5%_**	**Ib_10%_**	**Ib_20%_**	**Ib_30%_**	**Ib_40%_**	**Ib_50%_**	**Ib_60%_**	**Ib_70%_**	**Ib_80%_**	**Ib_90%_**	**Ib_99%_**
pH (Unit)	4.03	4.04	4.11	4.13	4.16	4.29	4.64	4.83	5.28	6.83	7.74	7.83
EC (mS/cm)	16.93	16.72	16.02	15.83	14.94	11.73	9.24	6.38	5.29	3.82	0.93	0.12

M_r_: Raw margin; Tr_x%_: percentage of Trebia’s bentonite; Ib_x%_: percentage of Iboughrdayn bentonite.

## Data Availability

The data presented in this study are available in this article.

## Sample Availability

Samples of the compounds are not available from the authors.
